# A comprehensive study capturing vision loss burden in Pakistan (1990-2025): Findings from the Global Burden of Disease (GBD) 2017 study

**DOI:** 10.1371/journal.pone.0216492

**Published:** 2019-05-03

**Authors:** Bilal Hassan, Ramsha Ahmed, Bo Li, Ayesha Noor, Zahid ul Hassan

**Affiliations:** 1 School of Automation Science and Electrical Engineering, Beihang University, Beijing, China; 2 School of Computer and Communication Engineering, University of Science & Technology Beijing, Beijing, China; 3 School of Computer Science and Engineering, Beihang University, Beijing, China; 4 Department of Psychology, International Islamic University, Islamabad, Pakistan; 5 Department of Pharmacology, Yusra Medical and Dental College, Islamabad, Pakistan; LV Prasad Eye Institute, INDIA

## Abstract

This study aims to provide estimates, trends and projections of vision loss burden in Pakistan from 1990 to 2025. Global Burden of Diseases, Injuries, and Risk Factors Study (GBD 2017) was used to observe the vision loss burden in terms of prevalence and Years Lived with Disability (YLDs). As of 2017, out of 207.7 million people in Pakistan, an estimated 1.12 million (95% Uncertainty Interval [UI] 1.07–1.19) were blind (Visual Acuity [VA] <3/60), 1.09 million [0.93–1.24] people had severe vision loss (3/60≤VA<6/60) and 6.79 million [6.00–7.74] people had moderate vision loss (6/60≤VA<6/18). Presbyopia was found to be the most common ocular condition that affected an estimated 12.64 million [11.94–13.41] people (crude prevalence 6.08% [5.75–6.45]; 61% female). In terms of age-standardized YLDs rate, Pakistan is ranked fourth among other South Asian countries and twenty-first among other 42 low-middle income countries (classified by World Bank), with 552.98 YLDs [392.98–752.95] per 100,000. Compared with 1990, all-age YLDs count of blindness and vision impairment increased by 55% in 2017, which is the tenth highest increase among major health loss causes (such as dietary iron deficiency, headache disorders, low back pain etc.) in Pakistan. Moreover, our statistics show an increase in vision loss burden by 2025 for which Pakistan needs to make more efforts to encounter the growing burden of eye diseases.

## Introduction

Pakistan is the sixth most populous country in the world with a population exceeding 207 million [1]. Still a developing country, Pakistan is listed in the category of “low-middle” income country according to World Bank income classification 2018 [2]. Consistent with World Bank development indicators, Pakistan has considerably improved in the healthcare sector over the past few decades. Life expectancy at birth was 60.1 years in 1990, which has increased to 66.5 in 2016. Similarly, under-5 mortality rate (per 1,000 live births) was 139 in the year 1990, which has reduced to 74.9 in 2017 [3]. However, more needs to be done to further improve the quality of life in Pakistan.

Visual acuity impairment severely degrades the quality of life and have more pronounced negative effects on people suffering from various other chronic health issues [4–6]. Globally, it has transformed into a major health problem. According to the statistics of the Global Burden of Disease (GBD) 2017 report, the third leading impairment was blindness and vision impairment that affected the greatest number of people, with 1·34 billion [95% UI 1·29–1·39] cases worldwide [7]. Globally as of 2017, 48.2 million people were blind, an additional 39.6 million had severe vision impairment, 279 million had moderate vision impairment, and 969 million had near vision impairment [8]. However, the burden of vision loss in Pakistan in the last one decade remained unclear.

In this study, we have comprehensively analyzed the vision loss burden due to numerous eye diseases in Pakistan from 1990 to 2017. Using the findings, we estimated the vision loss burden in 2025. We also quantified all causes leading to blindness and vision impairment in Pakistan and compared them with other South Asian countries and 42 low-middle income countries [2] using the GBD 2017 study.

## Previous work

National studies on blindness and its causes in Pakistan prior to this study are extremely limited. Until 1980, there was no data available to determine the prevalence and causes of blindness and visual impairment. First national survey was conducted between 1987–1990 by M. S. Memon [9] to estimate the prevalence and causes of blindness. They examined a total of 29157 subjects all over Pakistan and reported total blind prevalence as 9.03%. Among the various conditions responsible for blindness, cataract (66.7%) was found to be the major cause of blindness. Another national survey was conducted between 2002–2004 and the findings were reported in the form of two articles by B. Dineen et al. in [10] and M. Z. Jadoon et al. in [11]. They determined the causes and prevalence of blindness and visual impairment in adults aged 30 years and older in Pakistan, respectively. They examined 16507 subjects in total. Cataract was reported as the most common cause of blindness (<3/60) and second most common cause of moderate visual impairment (<6/18 to ≥6/60) after refractive error. The crude prevalence of blindness was 3.4% and severe vision impairment (<6/60) was 4.9%. 14.3% of the subjects presented a visual acuity <6/18 but ≥3/60 in the better eye. In 2005, R. Bourne et al. examined a total of 22600 subjects; including children aged 10 to 15 years and adults aged ≥30 years to determine prevalence rates and causes of blindness and low vision in Pakistan. They reported refractive error as the main cause of <6/12 and <6/18 visual acuity followed by cataract [12].

In addition, few regional/ local studies were also conducted on blindness and its causes. K. Ahmad et al. examined 1106 subjects, aged ≥40 years to determine the prevalence and causes of blindness and visual impairment in [13]. The study was conducted in the region of Budni, Peshawar, Pakistan. Out of total 1106 subjects, 21 were blind, 27 had severe visual impairment (<6/60–3/60) and 62 had visual impairment (<6/18–6/60). Men, as compared to women, had a higher prevalence of blindness, but they had a lower prevalence of severe visual impairment and visual impairment. Moreover, cataract was found to be the leading cause of blindness and low vision. In [14], K. M. Anjum et al. examined 1600 subjects, aged >50 years in Orakzai Agency, Pakistan, to estimate the rate, coverage and visual outcome of cataract surgery. The authors concluded that cataract was the leading cause of blindness in 82.4% of all blind cases and women, when compared with men, had a higher prevalence of cataract. S. Haider et al. [15] examined 1600 subjects aged ≥50 years to conduct rapid assessment of cataract surgery in Chakwal District, Pakistan. They concluded cataract being the major cause of bilateral blindness (VA < 3/60) in 46.5% of the total cases. S. P. Shaikh et al. [16] investigated the eye diseases pattern and prevalence in children aged 5 to 15 years. They examined a total of 5110 subjects at Bazzertaline Area, South Karachi, Pakistan in 2003. They reported 0.27% prevalence of bilateral blindness, with cataract being the major cause and 2.2% prevalence of low vision, with uncorrected refractive error being the leading cause. Moreover, they reported 1.72% higher visual impairment in girls as compared to boys.

All these previous studies were conducted more than a decade ago with certain limitations. Previous studies evaluated the burden of vision loss in Pakistan in terms of prevalence of eye diseases alone. We used both prevalence and YLDs to investigate the vision loss burden in our study. This enabled us to compare the burden of vision loss in Pakistan with those in other countries and other diseases in a more detailed manner. Moreover, the aim of this proposed national-level analysis is to present the comparative quantification of the burden of blindness and vision impairment along with its causes during 1990 to 2017. We have also projected the burden of blindness till 2025. This study is expected to yield important findings in accessing the burden of blindness and its causes in Pakistan over the years and might be helpful for future resource allocation. The previous studies conducted on blindness and visual impairment in Pakistan are summarized in Table 1.

**Table 1:** Summary of past studies conducted in Pakistan on blindness and its causes.

Reference	Coverage	Year	Age Range	Total Subjects	Urban/ Rural	Core VA level	Remarks
[9]	National	1987–1990	Anyone with VA <6/18 or any ocular abnormality	29157	Both	Blind (VA <3/60)	This study had some methodological limitations, considered only one VA level. Results were not age and gender specified.
[10], [11]	National	2002–2004	≥30 years	16507	Both	Blind (VA <3/60)	This study conducted perimetry on selected subjects only, which means that blind cases due to visual field defects alone such as glaucoma or retinitis pigmentosa would have been underreported. Also, lack of temporality of risk factor data was another limitation in this study.
						MVI (VA <6/18 to ≥6/60)	
						SVI (VA <6/60)	
						VA <6/18 but ≥3/60	
[12]	National	NS	10 to 15 years and ≥30 years	22600	Both	Blind (VA ***<***3/60)	This study reported some useful findings at that time. However, results were not age and gender specified.
						SVI (VA ***<***6/60 to 3/60)	
						MVI (VA ***<***6/18 to 6/60)	
						NN (VA ***<***6/12 to 6/18)	
						N (VA 6/12 or better)	
[13]	Local: Budni, Peshawar	1998	≥40 years	1106	Rural	Blind (VA *<*3/60)	Authors reported the prevalence of blindness and low vision by age-group, gender, occupation and based on cataract history. However, the reasons for insufficient number of cataract surgeries remained unclear.
						SVI (VA *<*6/60–3/60)	
						VI (VA *<*6/18–6/60)	
[14]	Local: Orakzai Agency	NS	>50 years	1600	Rural	Blind (VA<3/60)	Authors reported prevalence of cataract blindness and outcomes of cataract surgeries in local settings. However, the findings were only limited to cataract and other blind and low vision cases were not considered. In [6], the results on cataract blindness for females were not presented age and gender-wise due to unavailability of Orakzai Agency’s female population data in 1998 Pakistan census.
[15]	Local: Chakwal District	NS	≥50 years	1600	Both	Blind (VA < 3/60)	
						SVI (VA < 6/60)	
[16]	Local: Bazzertaline Area, South Karachi	2003	5 to 15 years	5110	Urban	Blind (VA < 3/60)	This study conducted perimetry on selected age-group subjects only. Secondly, being a population based cross-sectional survey lack of temporality of risk factor data was another limitation in this study.
						Low Vision	

NS = Not Specified, VA = Visual Acuity, MVI = Moderate Vision Impairment, SVI = Severe Vision Impairment, NN = Near Normal, N = Normal.

## Methods

### Overview

In our study, we used GBD 2017 data (http://ghdx.healthdata.org/gbd-results-tool) provided by The Institute for Health Metrics and Evaluation (IHME) for analyzing the vision loss burden in Pakistan from 1990 to 2017. The Global Burden of Diseases, Injuries, and Risk Factors is a detailed study of health loss capturing various diseases and injuries, their occurrence, prevalence and severity at a global level. The GBD 2017 study results were collected from 195 countries based on 354 causes and 3484 sequelae. 68781 data sources, such as extensive literature study, hospital and clinical data, surveillance and survey data from various sources, inpatient and outpatient medical records were used in total to compile these results. This comprehensive study was a reassessment to incorporate newly collected data to the previous GBD studies. GBD Results Tool provides the details of different risk factors, causes and impairments related to health in terms of deaths, Disability-Adjusted Life Years (DALYs), Years Lived with Disability (YLDs), Years of Life Lost (YLLs) and prevalence via age, year, gender and location. DisMod-MR 2.1, a Bayesian meta-regression tool, is used as the main technique to estimate these metrics for each health loss condition [7].

### GBD vision impairment data, assumptions and data adjustment

GBD collected the overall vision impairment data using representative population-based studies (such as peer-reviewed publications, grey literature, surveys etc.) conducted on measuring visual acuity. GBD also extracted data from the United States National Health and Examination Surveys (NHANES), World Health Organization (WHO) Studies on Global Ageing and Adult Health (SAGE), World Health Surveys (WHS), the Surveys of Health, Ageing, and Retirement in Europe (SHARE) and the Multi-Country Survey Study on Health and Responsiveness (MCSS). Additionally, they extracted the Rapid Assessment of Avoidable Blindness (RAAB) repository, vision impairment database in developing countries. Appendix 1 in [7] contains the exact search string used by GBD for extracting the vision impairment literature. Data that aligned with Snellen scale visual acuity levels was only considered, excluding the studies which did not access “presenting” or “best-corrected” vision. Furthermore, the studies presenting vision loss by cause were also considered to estimate the prevalence of vision loss due to various causes such as cataract, glaucoma, macular degeneration etc.

After retrieving data from all these sources GBD made certain assumptions and adjustments to the collected raw data for extrapolating the results; (1) In case where studies did not report separate blind and visual acuity severity estimates, GBD used linear regression method to predict ratios on age from studies that reported vision loss data by each severity, (2) In case where studies only reported best-corrected vision impairment estimates and did not report Presenting Vision Impairment (PVI) estimates, GBD used linear regression of logit-transformed prevalence of PVI with age, super-region random effects, fixed effects on best-corrected vision impairment and per capita Lag-Distributed Income (LDI) to predict PVI estimates for studies not reporting PVI data points explicitly. In DisMod-MR 2.1 model, these estimated data points were labeled with a study-level covariate which increased the standard error, (3) In case where estimated data points traversed more than twenty years of age, GBD used age-split algorithm to split the data points over five-year age groups by applying the age-pattern of the super-region. Appendix 1 in [7] explains the detailed modelling strategy for estimating severity-specific and cause-specific vision impairment.

### Uncertainty analysis

In GBD 2017 study, non-reference data types were multiplied by the exponentiated predictions from respective penalized spline regressions to crosswalk non-reference data types to reference data types. After data adjustments, uncertainty was accounted for using the Eq (1):

$$\varepsilon_{a}=\sqrt{\left({\varepsilon_{m}}^{2}.{\varepsilon_{s}}^{2})+({\varepsilon_{m}}^{2}.{\mu_{s}}^{2})+({\varepsilon_{s}}^{2}.{\mu_{m}}^{2}\right)}$$ (1)

Where *ε_a_* is the standard error of the adjusted non-reference data point, *ε_m_* is the exponentiated crosswalk prediction and *ε_s_* is the standard error the non-reference data point. *μ_s_* is the mean of the non-reference data point and *μ_m_* is the exponentiated crosswalk predictions from the penalized spline regressions.

Uncertainty Interval (UI) in GBD 2017 study was estimated by bootstrapping with 1000 samples. The distribution of each step was stored in 1000 draws, which was later used in computation process of every other step. For determining the distribution, sampling error of data inputs and uncertainties of the disability weights, severity distributions and the model coefficients were used. Mean estimate across 1000 draws was used for computing final estimates and based on the 25^th^ and 975^th^ ranked value across all 1000 draws, the 95% UI was determined. The detailed explanation on how uncertainty is accounted for in GBD study is mentioned in [7].

### GBD analytic model framework

The analytical modelling framework with major estimation components used by GBD to compile and estimate the results is explained step-by-step as following: GBD first takes the raw data sources in the form of community surveys, national surveys, case notifications, surveillance data, outpatient and Inpatient hospital data etc. In the next step, GBD performs certain data adjustments on the compiled raw data by applying age-sex splitting, adding study-level covariates, cause of death and demographic inputs and making other adjustments to the input data. Analytical DisMod-MR 2.1 estimation model along with injury modeling strategy and alternative modelling strategies are applied to the adjusted data, followed by impairments and underlying causes estimation, severity distribution, disability weights and comorbidity correction, to calculate the final burden estimates for each disease and injury by age, sex, year and country. The detailed explanation on GBD analytical modelling framework is shown in [7].

#### DisMod-MR 2.1 estimation model

GBD 2017 study used Bayesian meta-regression method DisMod-MR 2.1 as the estimation method. Before GBD 2010 study, most relevant single data source of a disease, location and time was used for nonfatal estimates in burden of disease assessments. DisMod-MR 1.0, the first version of DisMod was used in GBD 2010 study to evaluate more detailed information on a disease considering age-groups and data from multiple sources. DisMod-MR 1.0 analytical tool accounted for uncertainty intervals using Bayesian statistical methods and produced world regions estimates by evaluating and pooling all available data. DisMod-MR 2.0 version was introduced in GBD 2013 study, with increased computational speed and efficient implementation of model using C++ by altering model specification using log rates instead of negative binomial model. Also, all disease parameters were presented at country level rather than regional. DisMod-MR 2.1 version was introduced in GBD 2015 study by rewriting the code in Python language to organize the flow of data and settings in five levels: global, super-region, region, country and subnational (where applicable) location. For GBD 2016 and 2017 study, the computational engine DisMod-MR 2.1 mostly remained unchanged. However, GBD included new subnational locations each year and introduced new age groups 80–84, 85–89, 90–94, and 95+ in GBD 2016 study and onwards. Appendix 1 in [7] shows the DisMod-MR 2.1 analytical cascade in detail.

DisMod-MR 2.1 likelihood estimation is either based on a Gaussian, log-Gaussian, Laplace or Log-Laplace function. However, log-Gaussian equation is used as default for estimation of data likelihood. More detailed explanation of DisMod-MR 2.1 estimation model along with the relevant formulas and equations are provided in [7].

### Method for forecasting prevalence beyond 2017

In our study, we used Auto-Regressive Integrated Moving Average (ARIMA) model to forecast burden of blindness and vision impairment from 2018 to 2025. ARIMA is specified by three main component parameters known as P, D and Q. Briefly, they are:

- P stands for Autoregression, represents the lag order i.e. the number of lag observations in the model.
- D stands for Integrated, represents the degree of differencing i.e. the number of times input raw observations are differenced, in order to make the model stationary.
- Q stands for Moving Average, represents the order of moving average i.e. the size of moving average window applied to lagged observations.

We used MATLAB R2017a software, with its built-in “estimate”, “arima” and “forecast” functions to forecast the burden. These functions automatically test the ARIMA model for all possible values of P, D and Q and in the process selects the best model with right values of these parameters to use, hence avoiding the manual process of hit and trial methods.

### Definitions

#### Prevalence and YLDs

We used prevalence and YLDs as measure metrics for analyzing the vision loss burden in Pakistan. Prevalence number represents the total disease cases that are reported in a designated population at a given time. YLDs means years of life lived with any sort of health-related disability over time. YLDs count is calculated by taking the product of disability weight and prevalence number, where disability weight indicates a number between 0 and 1 representing the health loss severity related to a particular disease. It is estimated by comparing cross-cultural and worldwide health issues. YLDs rate is expressed as a rate per 100K and measured by dividing the total number of deaths due to a certain disease by the relevant population of that region. In this study, we quantified our results over time and with age (all-age, age-standardization and different age groups). Age-related diseases are often misrepresented due to the over or under representation of different age structures, therefore, age-standardization indicator is used to compare populations with different age groups [7].

#### Vision impairment categories and eye diseases

The GBD 2017 study considered presenting distance visual acuity <6/18 as vision impairment and listed it in following six different categories based on distance vision: Near Vision Loss (NVL), Mild Vision Loss (mVL), Moderate Vision Loss (MVL), Severe Vision Loss (SVL), Monocular Vision Loss (MonoVL) and Blindness. Table 2 defines the presenting distance visual acuity of each category in the better eye. Further, it has reported the following seven eye diseases: Trachoma, Glaucoma, Cataract, Macular Degeneration (Macular Edema, Dry or Wet Age-Related Macular Degeneration (ARMD) and other macular pathologies), Refraction Disorders (RD), Presbyopia and Other Vision Loss (OVL) [8]. A total of 57 eye conditions are categorized under the broad term of OVL [17].

**Table 2:** Vision impairment categories with corresponding visual acuity levels.

*Category*	*Case Definition* (Presenting distance VA* *in the better eye)*
*Near Vision Loss*	Near VA <6/18 distance equivalent
*Mild Vision Loss*	VA <6/12 but 6/18 or better
*Moderate Vision Loss*	VA <6/18 but 6/60 or better
*Severe Vision Loss*	VA <6/60 but 3/60 or better
*Monocular Vision Loss*	Blind in one eye and has difficulty judging distances
*Blindness*	VA <3/60 or <10% visual field around central fixation

VA = Visual Acuity

*modelled according to Snellen chart

## Results

### Crude and age-standardized prevalence

We used Pakistan 6^th^ national population and housing census data acquired from Pakistan Bureau of Statistics [1] website to estimate crude numbers affected and age-standardized prevalence of vision loss burden in 2017. Out of the 207.7 million total population of Pakistan in 2017, an estimated 21.78 million [95% UI 20.67–22.98] were affected from blindness and vision impairment (all-age crude prevalence 10.48% [95% UI 9.95–11.06]; 57% female). The age-standardized prevalence was 15.38% [95% UI 14.63–16.26]. Table 3 shows the detailed crude and age-standardized prevalence of ocular diseases and vision impairment categories in Pakistan for year 2017.

**Table 3:** Crude and age-standardized prevalence of vision loss burden in Pakistan in year 2017.

Population (million)	Name		All Ages		Age-Standardized	
			Number (million)	M:F	Number (million)	M:F
			*Prevalence (%)*	(%)	*Prevalence (%)*	(%)
Total: 207.7 Male: 106.4 Female:101.3	Eye Diseases	Glaucoma	0.07 (0.05–0.08)	51:49	0.14 (0.12–0.17)	51:49
			***0*.*03 (0*.*03–0*.*04)***		***0*.*07 (0*.*06–0*.*08)***	
		Cataract	2.52 (2.21–2.88)	46:54	5.36 (4.73–6.09)	46:54
			***1*.*21 (1*.*06–1*.*39)***		***2*.*58 (2*.*28–2*.*93)***	
		Age-related macular degeneration	0.05 (0.04–0.06)	50:50	0.10 (0.08–0.13)	51:49
			***0*.*02 (0*.*02–0*.*03)***		***0*.*05 (0*.*04–0*.*06)***	
		Refraction disorders	4.79 (4.20–5.45)	50:50	5.88 (5.17–6.65)	52:48
			***2*.*31 (2*.*02–2*.*62)***		***2*.*83 (2*.*49–3*.*20)***	
		Presbyopia	12.64 (11.94–13.41)	39:61	18.30 (17.32–19.44)	42:58
			***6*.*08 (5*.*75–6*.*45)***		***8*.*81 (8*.*34–9*.*36)***	
		Trachoma	0.15 (0.10–0.23)	46:54	0.34 (0.22–0.54)	46:54
			***0*.*07 (0*.*05–0*.*11)***		***0*.*16 (0*.*11–0*.*26)***	
		Other vision loss	0.53 (0.46–0.61)	50:50	0.78 (0.67–0.89)	52:48
			***0*.*26 (0*.*22–0*.*29)***		***0*.*37 (0*.*32–0*.*43)***	
	Vision Impairment Category	Near Vision Loss	12.64 (11.94–13.41)	39:61	18.30 (17.32–19.44)	42:58
			***6*.*08 (5*.*75–6*.*45)***		***8*.*81 (8*.*34–9*.*36)***	
		Mild vision loss	0.04 (0.01–0.08)	52:48	0.04 (0.01–0.08)	52:48
			***0*.*02 (0*.*01–0*.*04)***		***0*.*02 (0*.*005–0*.*04)***	
		Moderate vision loss	6.79 (6.00–7.74)	49:51	9.56 (8.40–10.93)	49:51
			***3*.*27 (2*.*89–3*.*73)***		***4*.*60 (4*.*04–5*.*26)***	
		Severe vision loss	1.09 (0.93–1.24)	49:51	1.89 (1.60–2.20)	48:52
			***0*.*52 (0*.*45–0*.*60)***		***0*.*91 (0*.*77–1*.*06)***	
		Blindness	1.12 (1.07–1.19)	45:55	2.06 (1.94–2.19)	44:56
			***0*.*54 (0*.*51–0*.*57)***		***0*.*99 (0*.*94–1*.*05)***	
		Monocular vision loss	0.10 (0.06–0.15)	51:49	0.11 (0.06–0.17)	51:49
			***0*.*05 (0*.*03–0*.*07)***		***0*.*05 (0*.*03–0*.*08)***	
	Total	Blindness and vision impairment	21.78 (20.67–22.98)	43:57	31.97 (30.40–33.78)	44:56
			***10*.*48 (9*.*95–11*.*06)***		***15*.*38 (14*.*63–16*.*26)***	

M = Male, F = Female

Data in parentheses are 95% uncertainty intervals.

#### Vision impairment categories

In terms of all-age prevalence, NVL (6.08% [5.75–6.45]; 61% female) was the main contributor towards total burden of vision loss in Pakistan. It was followed by MVL (3.27% [2.89–3.73]; 51% female), ranking second out of the six categories of vision impairment. Both blindness (0.54% [0.51–0.57]; 55% female) and SVL (0.52% [0.45–0.60]; 51% female) remained the third most common cause, followed by MonoVL (0.05% [0.03–0.07]; 49% female) and then mVL (0.02% [0.01–0.04]; 48% female) with the least prevalence in 2017.

#### Eye diseases

With over 12.64 million [95% UI 11.94–13.41] all-age cases reported (crude prevalence 6.08% [95% UI 5.75–6.45]; 61% female), presbyopia remained the leading ocular disorder in Pakistan. RD (2.31% [2.02–2.62]; 50% female) was found to be the second most common disorder, followed by cataract (1.21% [1.06–1.39]; 54% female), OVL (0.26% [0.22–0.29]; 50% female) and then by trachoma (0.07% [0.05–0.11]; 54% female). Glaucoma (0.03% [0.03–0.04]; 49% female) and ARMD (0.02% [0.02–0.03]; 50% female) remained the least common eye diseases in Pakistan for year 2017.

### 1990–2017 trends of vision impairment categories and eye diseases

In the vision impairment categories, NVL was the main contributor towards burden of vision loss in Pakistan from 1990 to 2017. In 1990, NVL contributed 52% to the total vision loss burden while the same amplified to 58% in 2017. It was followed by MVL, ranking second out of the six categories of vision impairment, with a contribution of 36% in 1990 and 31% in 2017. Both, blindness and SVL showed a negligible variation from 1990 to 2017 and remained the third more common category of vision loss burden. In 1990, blindness was 6% and SVL was 5%, while each of these categories contributed 5% in 2017. The all-age prevalence of MonoVL and mVL increased slightly over time during 1990 to 2017, but both contributed less than 1% to the overall burden of vision loss in Pakistan.

**Fig 1:**
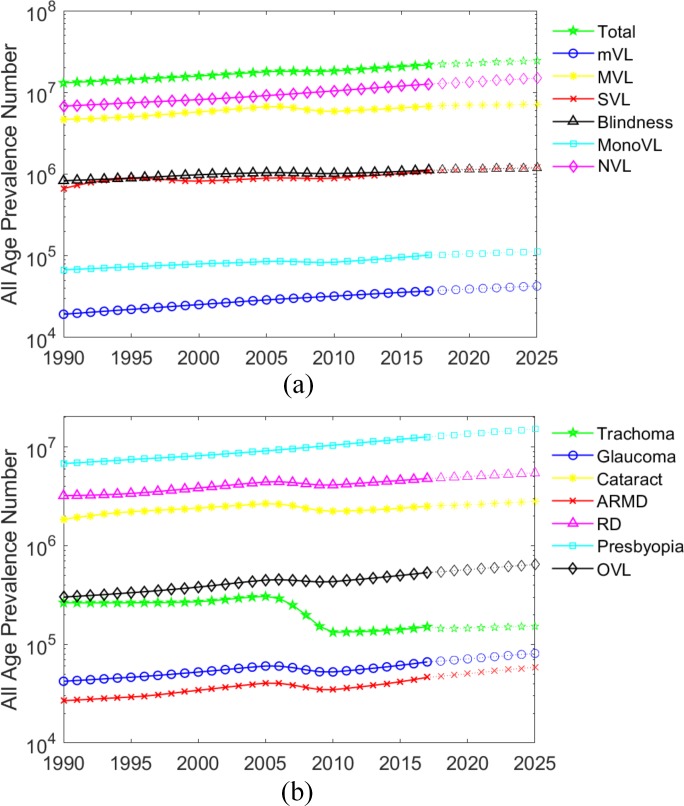
All-age prevalence number (1990–2025) for (a) blindness and vision impairment severity (b) various eye diseases.

**Fig 2:**
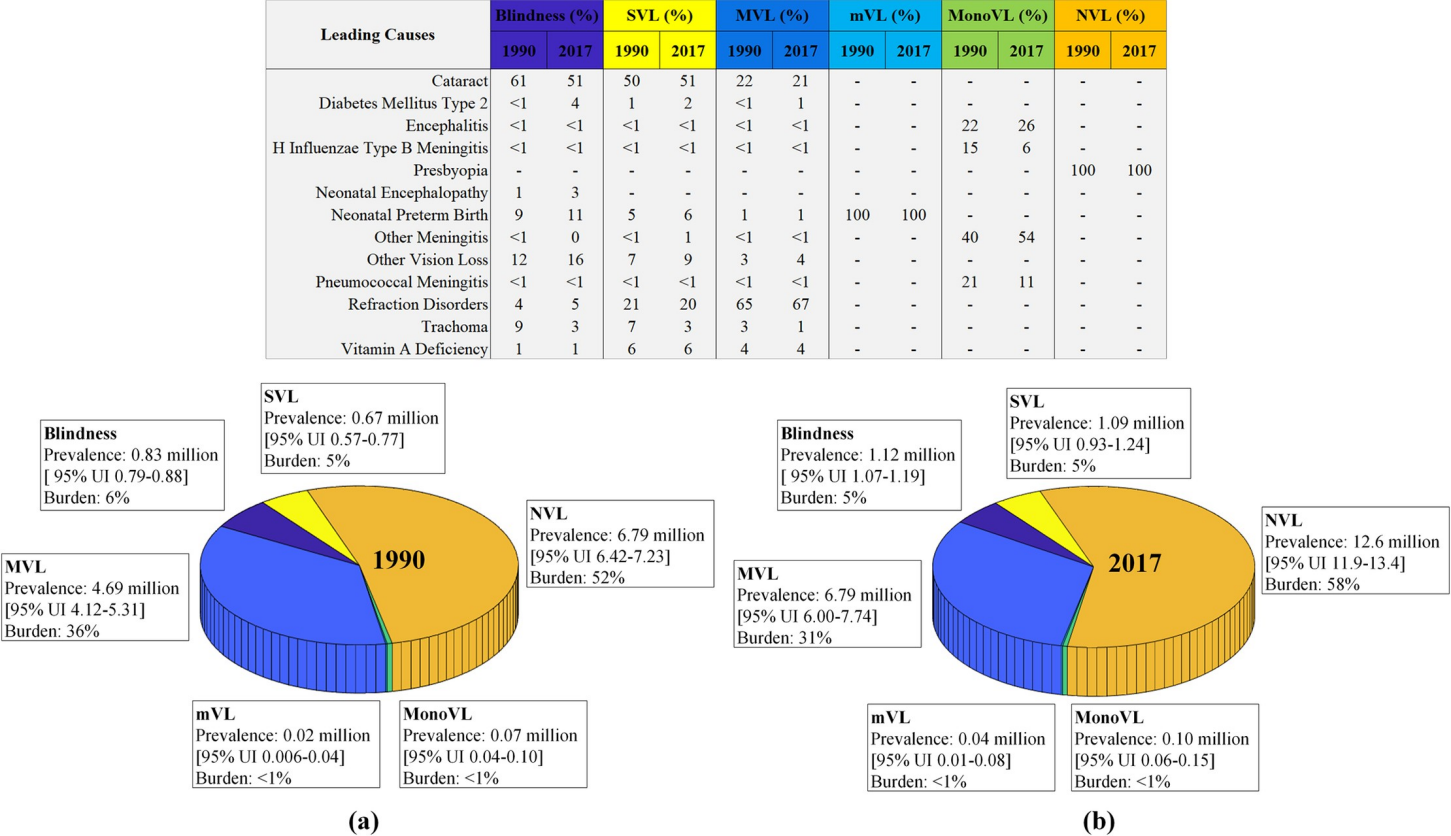
Distribution of blindness and vision impairment severity with leading causes for year (a) 1990 (b) 2017.

In relation to eye diseases, presbyopia persisted as the leading ocular disorder in Pakistan from 1990 to 2017, followed by RD being the second most common disorder. The all-age prevalence for both these diseases increased steadily over this period. The third more common eye disease was found to be cataract, followed by OVL and then by trachoma. The all-age prevalence of OVL showed a slight increase over time whereas, the numbers of trachoma fell significantly after 2005 until 2017. Glaucoma showed a steady rise in all-age prevalence from 1990 to 2017 followed by ARMD, which also increased considerably over time. The trends of vision loss categories and eye diseases can be observed in Fig 1 and Fig 2 in more detail.

### Projections of vision loss burden beyond 2017

It can be observed that the total blindness and vision impairment all-age prevalence increased steadily from 1990 to 2017 and the same statistics are projected with a rise in 2025. In relation to eye diseases, the probable statistics of 2025 show an increase in all the eye diseases except trachoma, which shows a decline in future. The projections of vision loss burden can be observed in more detail in Fig 1.

### Leading causes of vision impairment

Our results indicated that the prevalence of NVL due to presbyopia was 100%. RD was found to be the most common cause of MVL, followed by cataract, vitamin A deficiency, OVL and neonatal preterm birth for both year 1990 and 2017, while diabetes mellitus type 2 emerged as a new leading cause in 2017 contributing 1% to the entire MVL burden in Pakistan. Cataract, with the contribution of more than 50%, was found to be the most common leading cause towards blindness and SVL cases for both year 1990 and 2017. Neonatal preterm birth turned out to be the leading cause in all the mVL cases whereas, meningitis with the percentage of more than 40, was the most dominant cause in MonoVL cases, followed by encephalitis for each year 1990 and 2017. With respect to prevalence percentage, the leading causes of vision impairment categories are shown in Fig 2.

### Age-specific and gender-specific prevalence of eye diseases

**Fig 3:**
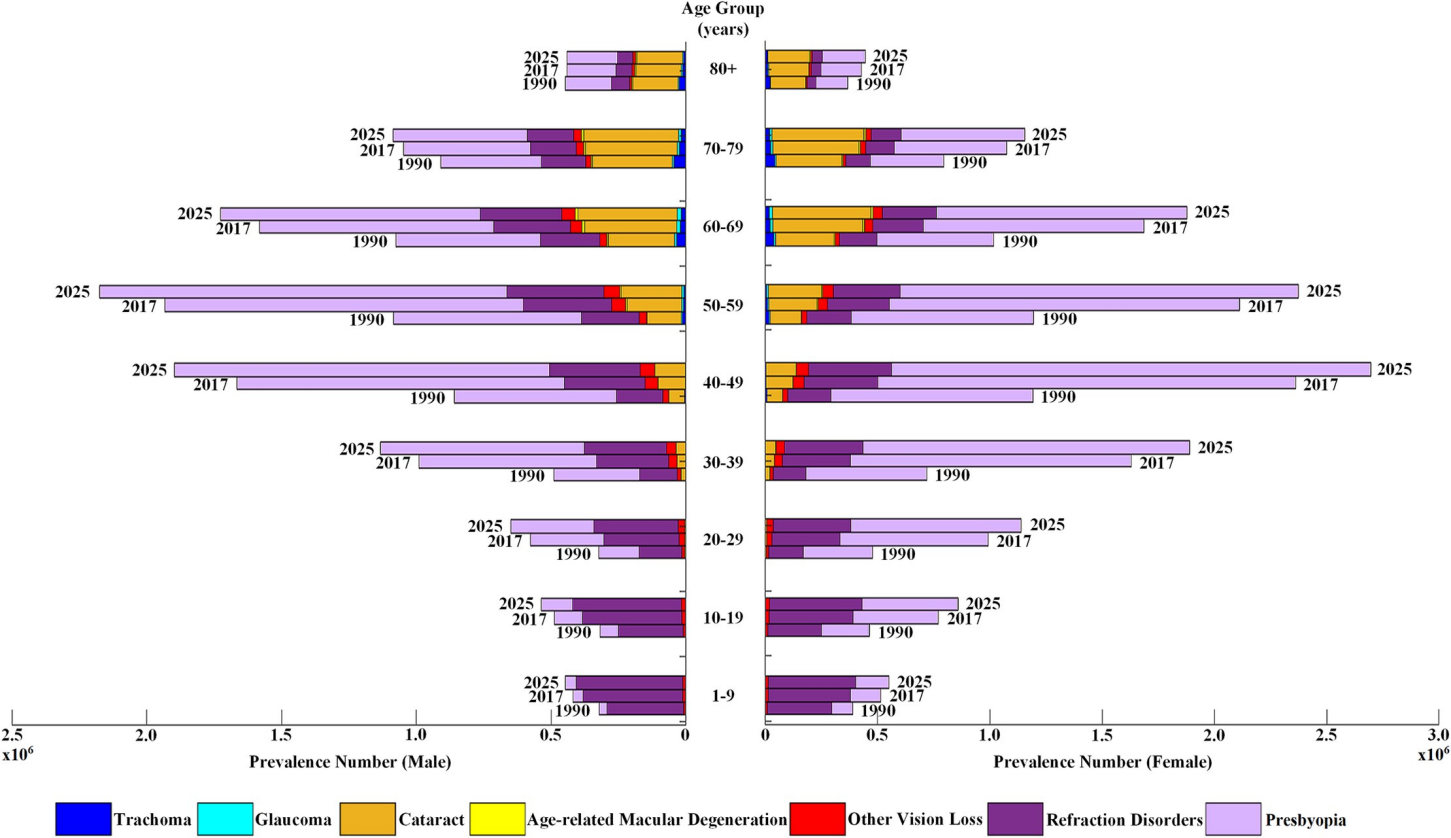
Age group and gender-wise prevalence of various eye diseases in year 1990, 2017 and 2025.

The burden of eye diseases increased between 1990 and 2017 among all age groups and genders, while the ranking of these diseases changed: presbyopia (first), RD (second) and cataract (third) were the most prevailing eye diseases both in male and female for aged 30–59 years, while for aged 60 years and above, cataract (second) replaced RD (third) in both genders. The occurring of trachoma, Glaucoma and ARMD notably increased for those aged 60 and above. Following the statistics from 1990 to 2017, the estimation in 2025 projects a rise in overall burden of eye diseases in Pakistan. Moreover, the prevalence number of females in ratio to males shows an increase in 2025. Fig 3 shows the detailed age group and gender-wise prevalence of eye diseases both in 1990, 2017 and estimating the burden in 2025 for Pakistan.

### Comparison of all-age prevalence with other South Asian countries

**Fig 4:**
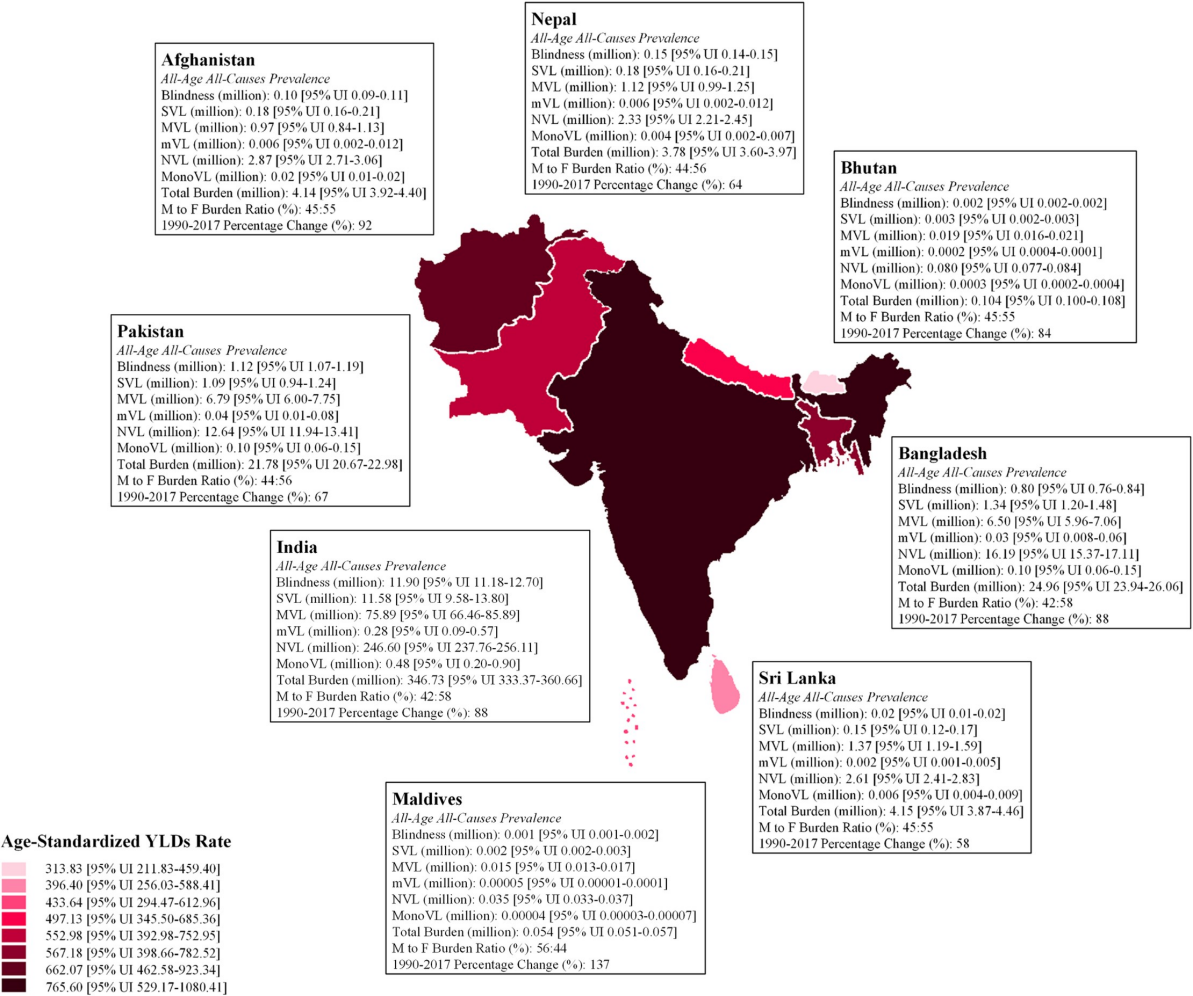
All-age prevalence and age-standardized YLDs rate for South Asian countries in year 2017.

Pakistan, with 21.78 million all-age prevalence of blindness and vision impairment is ranked third among other South Asian countries after India and Bangladesh. While, in terms of age-standardized YLDs rate, Pakistan is ranked fourth with 552.98 YLDs [95% UI 392.98–752.95] per 100,000. India is ranked on top with 765.60 YLDs [95% UI 529.17–1080.41], followed by Afghanistan and then by Bangladesh. Maldives having 0.054 million all-age prevalence had the least burden of blindness and vision impairment while, Bhutan with 313.83 YLDs [95% UI 211.83–459.40] per 100,000, is ranked last in terms of YLDs rate. The overall percentage change of vision loss burden in Pakistan during 1990 to 2017 was found to be 67%, which was the third least increase of burden in this period after Sri Lanka (58%) and Nepal (64%). All South Asian countries except Maldives showed higher prevalence of vision impairment in females. Presbyopia, RD and cataract remained the leading eye disorders in all the South Asian countries including Pakistan. Table 4 and Fig 4 give the detailed comparison of blindness and vision impairment of Pakistan with other South Asian countries.

**Table 4:** Age-specific vision loss burden with leading causes in South Asian countries in year 2017.

*Countries*	*Age-specific Vision Loss Burden (Prevalence %)*					*All-Age Leading Causes (%)*			Rank*
	1–4 years	5–14 years	15–49 years	50–69 years	70+ years	Presbyopia	RD	Cataract	
*Afghanistan*	3	10	53	23	11	69	18	5	2
*Bangladesh*	1	4	36	39	20	65	18	12	3
*Bhutan*	1	4	45	35	15	77	14	5	8
*India*	1	3	38	41	17	71	15	10	1
*Maldives*	1	4	44	33	18	65	19	10	6
*Nepal*	1	4	36	41	18	62	20	10	5
***Pakistan***	**2**	**7**	**43**	**34**	**14**	**58**	**22**	**12**	**4**
*Sri Lanka*	1	3	27	45	24	63	19	12	7

RD = Refraction Disorders

*Rank in terms of age-standardized Years Lived with Disability (YLDs) rate per 100K.

### Standing of Pakistan among other 42 low-middle income classified countries by World Bank

We found that all-age YLDs count increased in the majority of the countries including Pakistan between 1990 and 2017, while Pakistan ranked 4^th^ among 42 countries in 2017. However, age-standardized YLDs rate (per 100K) of Pakistan showed the 8^th^ highest decrease between 1990 and 2017, and as a result improved its position to 21^st^ rank among other listed countries in 2017. Presbyopia, RD and cataract remained the leading causes of visual impairment in all 42 countries including Pakistan in 2017, with more than 90% contribution to the overall burden of vision loss. Additionally, the prevalence ratio remained more in females in all countries including Pakistan except Papua New Guinea and Vanuatu. Table 5 gives a detailed comparative analysis of vision loss burden in Pakistan with other 42 low-middle income classified countries by World Bank in terms of age-standardized YLDs rate and all-age YLDs count between 1990 and 2017, and prevalence percentage for leading causes of visual impairment in 2017.

**Table 5:** Age-standardized YLDs rate (per 100K) and all-age YLDs count for vision loss burden between 1990 and 2017 in 42 low-middle income classified countries by World Bank, with leading causes in 2017.

*Countries*	*Age Standardized*				*All Ages*				*Causes for Vision Impairment in 2017* *(Prevalence %)*				
	1990	2017		% Change (1990–2017)	1990	2017		% Change (1990–2017)					
	YLD Rate (Per 100k)	YLD Rate (Per 100k)	Rank		YLD Count (100s)	YLD Count (100s)	Rank		P	RD	Cataract	All Other	M:F
*Angola*	751	638	11	-15	419	993	16	137	86	9	3	2	1:1.29
*Bangladesh*	672	567	18	-16	4031	7347	5	82	66	19	12	3	1:1.42
*Bhutan*	394	314	42	-20	13	23	37	77	79	14	5	2	1:1.22
*Bolivia*	716	577	16	-19	289	544	23	88	75	14	8	3	1:1.01
*Cambodia*	907	704	6	-22	511	847	20	66	59	23	14	4	1:1.35
*Cameroon*	631	534	23	-15	361	832	21	130	78	14	5	3	1:1.25
*Cote d'Ivoire*	794	688	8	-13	474	962	17	103	80	11	5	4	1:1.16
*D.R. Congo*	720	678	10	-6	1544	3204	10	108	88	8	3	1	1:1.23
*Djibouti*	593	499	25	-16	14	37	36	164	78	13	5	4	1:1.14
*Egypt*	814	623	13	-23	2844	4329	7	52	72	17	6	5	1:1.09
*El Salvador*	704	553	20	-21	254	324	27	28	74	16	8	2	1:1.48
*Micronesia*	446	409	37	-8	3	3	42	0	66	24	7	3	1:1.10
*Georgia*	427	415	35	-3	255	213	30	-16	67	14	17	2	1:1.32
*Ghana*	580	489	26	-16	496	961	18	94	72	19	6	3	1:1.26
*Honduras*	635	515	24	-19	177	363	26	105	77	16	5	2	1:1.33
*India*	815	766	3	-6	45102	88568	1	96	73	15	10	2	1:1.41
*Indonesia*	804	678	9	-16	9237	14962	2	62	76	9	12	3	1:1.11
*Kenya*	521	477	28	-8	645	1417	14	120	79	12	6	3	1:1.35
*Kiribati*	472	445	30	-6	2	4	41	100	66	25	6	3	1:1.25
*Lesotho*	628	575	17	-8	75	82	33	9	84	9	4	3	1:1.36
*Mauritania*	1092	920	1	-16	137	226	29	65	81	10	6	3	1:1.00
*Moldova*	443	427	33	-4	196	212	31	8	65	20	10	5	1:1.21
*Mongolia*	428	401	38	-6	59	102	32	73	64	22	11	3	1:1.10
*Morocco*	574	457	29	-20	1000	1520	12	52	84	8	6	2	1:1.43
*Myanmar*	1005	753	4	-25	2623	3404	9	30	41	29	25	5	1:1.28
*Nicaragua*	667	549	22	-18	147	284	28	93	77	14	6	3	1:1.31
*Nigeria*	830	702	7	-15	4512	7817	3	73	70	16	9	5	1:1.26
***Pakistan***	**703**	**553**	**21**	**-21**	**4742**	**7435**	**4**	**57**	**61**	**23**	**12**	**4**	**1:1.27**
*Papua New Guinea*	771	723	5	-6	177	398	24	125	56	30	9	5	1:0.95
*Philippines*	687	595	15	-13	2653	4808	6	81	87	8	3	2	1:1.07
*Sao Tome and Principe*	745	618	14	-17	6	8	39	33	74	14	8	4	1:1.20
*Solomon Islands*	472	443	31	-6	9	18	38	100	65	27	6	2	1:1.02
*Sri Lanka*	473	396	39	-16	602	941	19	56	64	19	13	4	1:1.25
*Sudan*	848	633	12	-25	982	1483	13	51	75	14	6	5	1:1.13
*Swaziland*	624	565	19	-9	27	42	35	56	85	9	3	3	1:1.36
*Timor-Leste*	1103	839	2	-24	42	75	34	79	51	28	14	7	1:1.05
*Tunisia*	662	483	27	-27	385	580	22	51	78	9	8	5	1:1.18
*Ukraine*	343	341	41	-1	2216	2228	11	1	71	15	10	4	1:1.80
*Uzbekistan*	449	421	34	-6	644	1068	15	66	64	22	12	2	1:1.09
*Vanuatu*	395	377	40	-5	4	8	40	100	66	26	6	2	1:0.99
*Vietnam*	612	431	32	-30	2835	3895	8	37	59	19	13	9	1:1.39
*Zambia*	481	412	36	-14	198	392	25	98	78	12	5	5	1:1.38

YLDs = Years Lived with Disability, P = Presbyopia, RD = Refraction Disorders, M = Male, F = Female.

### Leading causes of health loss in Pakistan between 1990 and 2017

Compared with 1990, the health loss burden increased in 2017 in Pakistan. All-age YLDs count of blindness and vision impairment increased by 55% in 2017, which is the tenth highest increase among other health loss causes. Whereas, in terms of age-standardized YLDs rate, blindness and vision impairment reduced by 21% in 2017, which is the fourth highest decrease among other health loss causes. Table 6 shows a detailed comparison of burden of blindness and vision impairment with other major health loss causes in Pakistan between 1990 and 2017.

**Table 6:** All-age YLDs count and age-standardized YLDs rate (per 100K) for leading causes of health loss in Pakistan between 1990 and 2017.

*CAUSES*	*ALL AGE*					*Age Standardized*				
	1990		2017		Percentage Change during 1990–2017 (%)	1990		2017		Percentage Change during 1990–2017 (%)
	YLD Count (100s)	Rank	YLD Count (100s)	Rank		YLD Rate (Per 100k)	Rank	YLD Rate (Per 100k)	Rank	
*Dietary iron deficiency*	12695	1	15096	1	19	1084	1	683	2	-37
*Headache disorders*	6102	2	13409	2	120	672	3	669	3	0
*Low back pain*	5368	3	12314	3	129	710	2	746	1	5
*Neonatal disorders*	3789	7	9369	4	147	289	12	389	10	35
*Depressive disorders*	4443	5	9319	5	110	556	5	529	5	-5
*Diabetes mellitus*	2702	13	8462	6	213	404	9	596	4	48
*Other musculoskeletal disorders*	3754	8	7795	7	108	492	7	471	7	-4
*Anxiety disorders*	3252	9	7077	8	118	349	10	353	11	1
*Age-related and other hearing loss*	3146	10	6144	9	95	462	8	450	9	-3
***Blindness and vision impairment***	**3862**	**6**	**5970**	**10**	**55**	**602**	**4**	**474**	**6**	**-21**
*Chronic obstructive pulmonary disease*	3108	11	5842	11	88	503	6	465	8	-8
*Diarrheal diseases*	2875	12	4994	12	74	254	13	236	14	-7
*Upper digestive system diseases*	1600	18	4340	13	171	190	17	239	13	26
*Drug use disorders*	1716	15	3876	14	126	193	16	188	16	-3
*Dermatitis*	2042	14	3842	15	88	162	18	162	19	1
*Neck pain*	1694	16	3765	16	122	241	14	242	12	0
*Oral disorders*	1524	20	3477	17	128	204	15	221	15	8
*Epilepsy*	1536	19	3207	18	109	141	23	149	21	6
*Vitamin A deficiency*	5129	4	3082	19	-40	303	11	115	29	-62
*Hemoglobinopathies and hemolytic anemias*	1690	17	2231	29	32	152	21	106	32	-30

YLDs = Years Lived with Disability

## Discussion

To the best of our knowledge, very few national studies have been conducted in the past to investigate the burden of vision loss in Pakistan. We used GBD 2017 data released on November 8, 2018 to conduct our study. The main contributions of our study are to investigate: 1) total burden of vision loss from 1990 to 2025, 2) burden of individual eye diseases from 1990 to 2025, 3) severity of vision loss burden in Pakistan between 1990 and 2017, 4) prevalence of eye diseases in relation to different age groups and gender in 1990, 2017 and 2025, 5) comparative analysis of vision loss burden in Pakistan among other South Asian countries and 42 low-middle income countries, and 6) comparison of vision loss burden with other major causes of health loss in Pakistan.

Our findings indicate that the burden of vision loss in Pakistan had been on the rise from 1990 to 2017 and is estimated to further increase at a steady rate by 2025, unless some major developments are made to control this burden. Among vision impairment categories, the combined crude prevalence of MVL and SVL in 2017 was 3.79% [95% UI 3.34–4.33], and the leading causes were RD (87%) and cataract (72%). The crude prevalence of blindness was 0.54% [0.51–0.57], with cataract (51%) being the leading cause. These findings are consistent with other global studies [18–19]. In relation to eye diseases, presbyopia, RD and cataract were the three dominating impairments in Pakistan, which is also consistent with other regional studies [20–24]. Due to ageing and population growth, the burden of most eye diseases increased between 1990 and 2017 and the same statistics are predicted to rise until 2025. It is concerning that for the past 27 years, the prevalence of presbyopia, refraction and macular disorders increased progressively whereas most of these pathologies are avoidable and treatable if diagnosed in time. Secondly, most of the presbyopia and RD can be corrected safely and with less expenses, more efforts should be made in time to avoid such cases. Furthermore, these disorders mostly fall under avoidable blindness therefore, additional priority should be accorded in this area. This also aligns with the initiative of “VISION 2020: the Right to Sight”, which aims to eliminate avoidable blindness by the year 2020 [25]. Additionally, cataract was found to be the third leading cause of vision loss burden, and the dominating cause of blindness and SVL in Pakistan with a contribution of more than 50%.

It is worth noting that Pakistan has well controlled the overall prevalence of blindness and vision impairment in comparison to the staggering increase in its population. According to the Pakistan’s National Census Report 2017, the population of Pakistan increased by 60% from 1998 to 2017 [1], whereas the total burden of vision loss (all vision impairment categories inclusive) increased by 43% during this time. Additionally, the burden due to blindness and SVL among other vision impairment categories mostly remained unchanged from 1990 to 2017 as shown in Fig 1A.

Even though the overall burden of impairment due to vision loss has significantly reduced by 21% among other health loss problems in Pakistan as shown in this study, the condition of health infrastructure is not very satisfactory in general. Specialized hospitals are numbered, there is a scarcity of basic health units or lack of newest health services at a wide scale. Also, the reach to the tertiary level health centers is mostly limited because the major proportion of the population dwells in rural areas, where the health infrastructure is considerably weak [26–28]. These very conditions are likewise applicable to the ophthalmology institutes in Pakistan. Lack of advanced surgical techniques, trained eye-care workforce, access to eye-care services and post-operative follow-ups are most likely reasons for cataract being the dominant cause of blindness and SVL in Pakistan between 1990 to 2017 [29]. Additionally, it is seen that the quality of cataract surgery in Pakistan is not up to the mark resulting in sizable blindness cases after surgery [30]. Furthermore, it was observed in our results that the prevalence of cataract in females was more as compared to males, this is presumably due to the factor of gender disparity in quantity of cataract surgery in Pakistan. However, one positive sign was the drastic decline in prevalence of trachoma after 2005.

Further, when we studied the burden of vision loss by gender and age group, it was observed that the burden of vision loss for male was most between aged 50–59 years as compare to all other age groups. Whereas, for female the maximum burden was between aged 40–49 years when compared with all other age groups. In the younger ages (aged 1–29 years), presbyopia and RD are more prominent among all other eye diseases. Cataract emerged in the age of 30 years and above among both genders, while trachoma and glaucoma appeared in the older ages (aged 60 years and above) among both genders. Since, major causes of blindness and vision loss are related to age such as glaucoma, macular degeneration and cataract, the ageing population adds a substantial weight to the overall burden of vision loss. Additionally, as mentioned above, life expectancy at birth has significantly improved in Pakistan since 1990, which may have resulted in higher prevalence for eye diseases of ageing and therefore, Pakistan’s national health policy should assign precedence to control these growing eye diseases. Among other South Asian countries, Pakistan ranked fourth in terms of age-standardized YLDs rate (per 100K) and third in terms of all-age prevalence of blindness and vision impairment in 2017. Further, in comparison to other 42 low-middle income countries, the vision loss burden in Pakistan has significantly reduced by 21% in terms of percentage change between 1990 and 2017 for age-standardized YLDs rate (per 100K). Pakistan has shown the 8^th^ highest decrease among other 42 countries despite all challenges, which is a satisfactory performance.

This study has several limitations. Although GBD 2017 study made efforts to collect data from all possible sources, the quality and quantity of available data are still limited, resultantly affecting the accuracy of estimated burden. GBD shows considerable heterogeneity in terms of data density. This may result in non-consistent correlation between available data and burden estimates. GBD uses Medical Expenditure Panel Survey (MEPS) by USA to estimate the global severity distributions of diseases in their study. This poses a limitation because the severities experienced in one population may not reflect the severity distributions worldwide and are most likely to change with location, age, time and treatment access. GBD heavily relies on clinical data records, with efforts to correct selection bias in clinical records, it uses representative studies as reference from USA and Taiwan claim records only. This poses limitations to the generalizability of these data adjustments. Moreover, GBD 2017 cause-list included 354 diseases/injuries to estimate impairments. However, this has led to inaccurate estimates for some impairments, for example, in our study mVL and MonoVL are specified by very few causes. This resulted in less estimates of these impairments when compared to MVL. As MVL had increased significantly, one would assume same for mVL and MonoVL. Therefore, there is a need to incorporate a greater level of detail in the GBD cause-list to estimate impairments with much more accuracy. The burden of vision loss for children younger than 15 years may be underestimated due to exclusion of certain rare ocular diseases such as congenital cataract and congenital glaucoma. Despite these many limitations, the GBD annual reports are very helpful and provide a standardize approach to evaluate and access the burden of various diseases across time and place.

In summary, this study illustrates the insights of vision loss burden in Pakistan, which mostly remained unclear in the past decade or more. As mentioned earlier, Pakistan has shown noteworthy improvement in restricting the burden over the last 27 years despite so many challenges its way. However, our statistics show a rise in vision loss burden in future for which Pakistan’s needs to make more efforts, such as according national priority to this cause by implementing effective policies and measures in the health sector, setting eye-care centers in remote areas, substituting trained eye-care workforce, and by facilitating ophthalmologists with state-of-the-art computer-aided diagnosis and detection infrastructure [31–32] to tackle the growing burden of vision loss.

## Supporting information

S1 Table Global Burden of Diseases (GBD), 2017 study data used in Fig 1A and Table 3. (XLSX)

S2 Table Global Burden of Diseases (GBD), 2017 study data used in Fig 1B and Table 3. (XLSX)

S3 Table Global Burden of Diseases (GBD), 2017 study data used in Fig 2. (XLSX)

S4 Table Global Burden of Diseases (GBD), 2017 study data used in Fig 3. (XLSX)

S5 Table Global Burden of Diseases (GBD), 2017 study data used in Fig 4. (XLSX)

S6 Table Global Burden of Diseases (GBD), 2017 study data used in Table 4. (XLSX)

S7 Table Global Burden of Diseases (GBD), 2017 study data used in Table 5. (XLSX)

S8 Table Global Burden of Diseases (GBD), 2017 study data used in Table 6. (XLSX)
